# The influence of soft wheat grain germination on its technological properties

**DOI:** 10.1371/journal.pone.0329975

**Published:** 2025-08-13

**Authors:** Darigash Shaimerdenova, Aigul Omaraliyeva, Baltash Tarabayev, Zhanar Chakanova, Damira Iskakova, Gaini Sarbassova, Maigul Kizatova, Sandugash Anuarbekova

**Affiliations:** 1 Department of Science, LLP “Research and Production Enterprise “Innovator”, Astana, Kazakhstan; 2 Technology of Food and Processing Industries, Saken Seifullin Kazakh Agrotechnical University, Kazakhstan; 3 Department of Pharmaceutical Technology, Kazakh National Medical University, Almaty, Kazakhstan; Institute of Genetics and Developmental Biology Chinese Academy of Sciences, CHINA

## Abstract

This study evaluates the impact of controlled germination on the technological properties of soft wheat grain (Tәuelsizdik variety) to determine its suitability for flour production. Grain was germinated for 24, 48, and 72 hours and incorporated into commercial flour at varying ratios (5%, 10%, and 15%) with gluten contents of 26%, 28%, and 30%. Technological properties including flour strength (W), water absorption capacity (WAC), and falling number (FN) were assessed using standard analytical methods and optimized through a Box–Behnken design with response surface modeling. Results revealed that increasing germination time significantly reduced gluten content, FN, and dough rheological parameters such as tenacity and elasticity, while increasing extensibility. Optimal technological performance was achieved with 29.8% gluten content, 24 hours of germination, and 8.2% germinated grain, yielding strong flour characteristics: W = 307.6 × 10 ⁻ ⁴ J, WAC = 54.9%, and FN = 180.4 s. The study highlights that, under optimal conditions, germinated soft wheat grain can be effectively utilized in flour production without compromising quality, offering a valuable strategy for processing sprouted grain resulting from unfavorable harvest conditions.

## Introduction

Wheat is one of the leading cereal crops and is considered a staple food for approximately 40% of the global population [1]. The International Grains Council (IGC) has revised its forecast for global wheat production in the 2024/25 season downward by 2 million tonnes, bringing the total to 796 million tonnes [2]. In contrast to this projection, global wheat consumption in the 2024–2025 season is expected to reach 804 million tones, which, along with carryover stocks, may result in a reduction of global ending stocks by 17 million tonnes [3].

In 2024, Kazakhstan harvested a record 26.5 million tonnes of grain (pre-cleaning weight), significantly exceeding the 17.1 million tonnes collected in 2023 [4]. With domestic demand at approximately 8 million tones [5], the country holds strong prospects in the international grain trade. However, climatic conditions in Kazakhstan’s grain-producing regions, particularly those cultivating soft wheat, significantly affect both the quality and quantity of the harvested crop. For instance, excessive rainfall during the 2023 harvest period led to a substantial decline in the technological properties of soft wheat grain. As a result, approximately 40% of the wheat delivered to grain elevators was classified as substandard due to the presence of sprouted kernels [6]. These climate-induced quality losses in grain production reflect a broader trend observed in Kazakhstani agriculture, where efforts to preserve productivity—whether in crops or livestock—are increasingly reliant on biotechnological innovations [7–9].

In this context, the investigation of the potential use of sprouted grain for technological purposes is of considerable interest. Grain germination is triggered by factors such as high humidity, frequent precipitation, and low temperatures occurring after the seeds reach physiological maturity but prior to harvest [10]. Germination is a complex physiological and biochemical process involving the activation of dormant tissues and the initiation of growth mechanisms. This process is influenced by numerous factors, the effects of which on grain quality and final product characteristics have been the subject of extensive research [1,11–15].

Pre-harvest sprouting (PHS) of soft wheat (*Triticum aestivum* L.) refers to the phenomenon in which physiologically mature grains begin to germinate while still on the spike prior to harvest. Bread produced from such grain tends to be porous, sticky, and lacking in color due to elevated activity of amylases, lipases, and proteases, which degrade starch, lipids, and proteins in the sprouting kernels [16]. For example, in China, the total area affected by PHS in certain years can reach approximately 25 million hectares—equivalent to 83% of the country’s wheat cultivation area—particularly in spring wheat-growing regions characterized by frequent rainfall and high humidity during the harvest period [17]. According to expert estimates, global economic losses from pre-harvest sprouting are as high as USD 1 billion annually [18].

It is widely acknowledged that the onset of PHS triggers a cascade of biochemical events resulting in the production of enzymes that degrade starch and protein, breaking them down to provide energy for germination. Premature sprouting is directly associated with reductions in both yield and product quality [19]. Yield loss is primarily attributed to a decrease in test weight, which is linked to endosperm degradation occurring in the early stages of germination [20]. The increase in enzymatic activity caused by sprouting can negatively affect the rheological properties of dough and the quality of the final baked product, as the germination process reduces the breadmaking performance of the flour [14]. A distinction is noted between PHS—characterized by highly heterogeneous germination patterns—and controlled germination of grain in experimental settings. Nevertheless, the latter has been widely used to investigate germination-induced changes [21–23]. For decades, researchers have studied the impact of PHS on the production of cereal-based products. Among the most effective approaches to mitigating losses caused by PHS is the selection and cultivation of wheat genotypes with inherent resistance to this phenomenon [24,25]. Such an approach makes it possible to prevent the deterioration of grain quality already at the stages of maturation and harvesting, particularly under conditions of elevated humidity. Nonetheless, in cases where pre-harvest sprouting has already occurred, an urgent task remains the development of technological strategies for processing sprouted grain while preserving its nutritional and functional properties, which constitutes the objective of the present study. To date, much of the current knowledge on pre-harvest sprouting has been derived from studies involving wheat germinated under controlled laboratory conditions, used as a proxy for field-sprouted grain. However, germination under controlled conditions typically results in significantly higher and more uniform enzymatic activity and degradation of key endosperm components [26]. According to Romano and Stevanato [27], differences in the degree of sprouting between field samples and laboratory-germinated specimens used in research may partly explain the inconsistencies observed across studies, and this factor should be taken into account.

Unlike several previous studies [28,29], the present study focuses on analyzing the changes in composite flour blends containing different proportions of germinated wheat grain. This approach enables not only the evaluation of the effects of germination but also the identification of optimal conditions for its rational technological application. Therefore, the present study examines the effects of controlled laboratory germination of soft wheat grain on its technological properties and explores its potential for further application.

## Materials and methods

### Materials

The study utilized soft wheat grain of the *Tәuelsizdik* variety from the 2024 harvest, cultivated in the Karaganda region of Kazakhstan, as well as commercial wheat flour derived from soft wheat, with the characteristics presented in Table 1.

**Table 1:** Characteristics of materials used in the study.

Parameters	Soft Wheat Grain (*Tәuelsizdik* variety)	Commercial Wheat Flour		
		Gluten content, %		
		26,0	28,0	30,0
Protein content (% by mass)	14,44 ± 0,05	12,8 ± 0,02	13,3 ± 0,02	14,5 ± 0,03
Fat content (% by mass)	2,07 ± 0,02	1,11 ± 0.1	1,52 ± 0,0	1,15 ± 0,04
Starch content (% by mass)	57,48 ± 0,03	68,9 ± 009	67,8 ± 0,2	70,6 ± 0,09
Moisture content (% by mass)	13,36 ± 0,02	13,4 ± 0,02	13,9 ± 0,02	13,7 ± 0,01

The data are presented as mean ± SE (standard error); the sample size was three (n = 3), and the level of statistical significance was set at ≤ 0.05.

No specific permits were required for the fieldwork conducted in this study, as the research activities were carried out on institutional land under the ownership of “Innovator”.

### Germination

The grain used in this study was germinated following a protocol modified by Ahmed, Ragab [30] and Cardone, D’Incecco [26]. Briefly, the wheat samples were rinsed with tap water and subsequently disinfected using a 1% aqueous sodium chloride (NaCl) solution for 30 minutes, followed by thorough rinsing with water until a neutral pH was achieved. After the final rinse, the wheat grains were soaked in water at a grain-to-water ratio of 1:2 for 24 hours at 20°C. The soaked grains were then evenly spread in germination trays, covered with a white, moistened cotton cloth, and left to germinate for 24, 48, and 72 hours at 25 ± 1°C and 85% relative humidity. The germinated wheat was dried in a convection oven at 50–55°C for 5 hours until a final moisture content of 9.0–12.0% was reached. The drying temperature regime for the germinated wheat (50–55°C) was selected to preserve enzymatic activity and prevent protein denaturation, which is critical for the subsequent analysis of the physicochemical and technological properties of the resulting flour.

### Flour preparation

For subsequent analyses, the germinated wheat was milled to a particle size of < 0.8 mm using a laboratory mill (Stegler LM-500, 28,000 rpm). The resulting flour was stored in glass containers at approximately 4°C until further testing. Flour blends were then prepared by mixing commercial wheat flour of varying gluten contents with germinated wheat flour in the following ratios: 95:5%, 90:10%, and 85:15%.

### Determination of gluten quantity and quality

The mass fraction and quality of gluten were assessed according to the method described in ISO 21415−1 [31].

### Determination of the falling number index

The Falling Number (FN) value of flour (Hagberg–Perten method) was determined in accordance with ISO 3093 [32], using a PCHP-7 device (with cooling) with a flour sample of 7 g (adjusted to 14% moisture content) suspended in 25 mL of water.

### Determination of physicochemical parameters

The moisture content of all materials and protein content in both grain and flour were measured according to the American Association of Cereal Chemists (AACC) protocol [33]. The starch content was determined using ISO protocol [34].

### Determination of water absorption capacity

The water absorption capacity (WAC) of wheat flour was determined using a consistograph, following the AACC protocol [35]. In this procedure, dough is prepared from wheat flour by adding a calculated amount of water, based on the initial moisture content of the flour, to achieve a constant level of hydration on a dry matter basis. During the mixing process, pressure on one side of the mixer is continuously monitored. The peak pressure recorded during mixing is used to calculate the water absorption of the flour sample at a specified dough consistency. In a subsequent test, conducted at the previously determined hydration level, the physical properties of the dough, including the WAC of the wheat flour, are evaluated. This method is applicable to all types of wheat flour.

### Data analysis

The results were expressed as the mean of three replicates ± standard deviation. Experimental data were processed using Microsoft Excel and MathCad software. The obtained data are presented at a confidence level of 95%. Statistical analysis was performed using two-way ANOVA. The optimization of the technological properties of wheat flour produced from germinated wheat seeds, aimed at evaluating the combined effects of germination parameters, was conducted using a three-level Box–Behnken design; with data analysis performed using the Statgraphics Centurion 19 software.

The controlled factors selected for the experimental design were: gluten content (x₁), germination time (x₂), and proportion of germinated grains (x₃). The experimental plan involved varying each factor at three levels in accordance with the Box–Behnken design. All other experimental conditions were kept constant. The response variables were:

where, y₁ – flour strength; y₂ – WAC; y₃ – FN.

The number of experimental factors was three, and a total of 15 experiments were conducted, including three replicates at the central point. The degrees of freedom for error were five.

## Results

### Alterations in the chemical composition of soft wheat grain during germination

**Fig 1:**
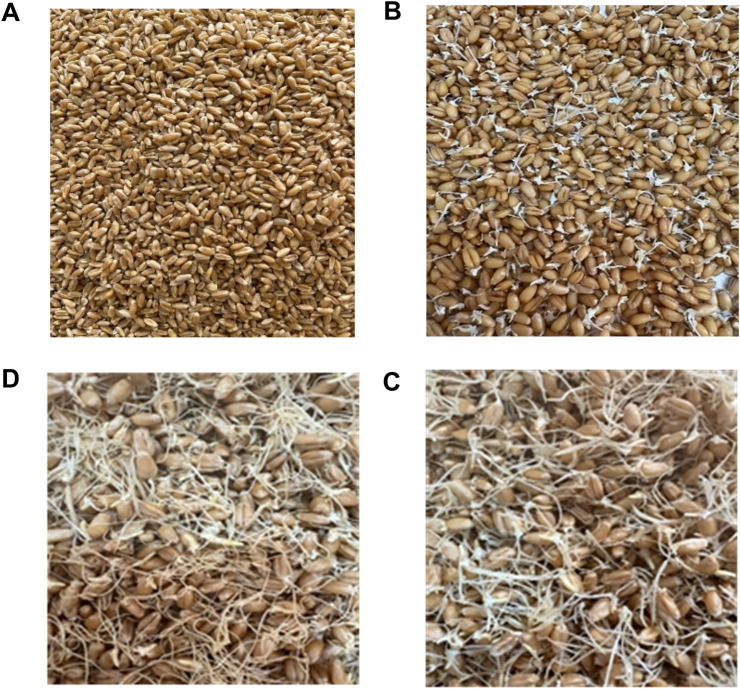
Initial soft wheat grain of the Tәuelsizdik variety. Initial Grain (A) and after 24 (B), 48 (C), and 72 (D) hours of germination.

External morphological alterations in *Tәuelsizdik* soft wheat grain from the 2024 harvest were observed as early as 24 hours after the onset of germination (Fig 1). At this point, the grains showed initial sprouting, while after 48 and 72 hours, the sprouts had developed substantially, exceeding the size of the grain itself.

During the early stage of grain germination (24 hours), a slight increase in crude protein content was observed—from 14.43% to 14.52% (Table 1). This effect is attributed to a combination of biochemical processes occurring in the grain under the influence of the enzymatic system. Initially, amylase activation leads to partial starch hydrolysis and a consequent reduction in carbohydrate content, resulting in a relative increase in protein content when calculated on a dry matter basis. Concurrently, protease activity initiates the mobilization of storage proteins and the synthesis of new enzymatic and structural proteins required for sprout development. In terms of starch content, an increase was noted after 24 hours of germination—from 57.48% to 59.54% S1 Table. However, by 72 hours, starch levels had decreased to 53.88%. This fluctuation is likely attributable to the initial quality of the grain; higher-quality grain tends to be more resistant to enzymatic degradation. The falling number (FN) index is directly influenced by alpha-amylase activity. In our experiments, the initial FN value of 233 seconds dropped sharply to 62 seconds after 24 hours of germination (Table 2).

**Table 2:** Parameters of the initial Tәuelsizdik wheat sample and during the germination process.

Parameters	Initial	Germination Time, hours		
		24	48	72
Moisture content, %	13,36 ± 0,03^a^	9,41 ± 0,12^c^	9, 43 ± 0,03^c^	9,55 ± 0,02^b^
Protein content, %	14,4 ± 0,12^с^	14,52 ± 0,08^a^	14,57 ± 0,05^a^	13,29 ± 0,05^b^
Gluten content, %	28,22 ± 0,09^c^	27,71 ± 0,13^b^	26,15 ± 0,01^b^	23,38 ± 0,09^a^
Starch content, %	57,48 ± 0,08^b^	59,54 ± 0,03^c^	56,67 ± 0,06^b^	53,88 ± 0,07^a^
Falling number, sec	233 ± 0,18^b^	62 ± 0,0^a^	60 ± 0,0^a^	60 ± 0,0^a^

The data are presented as mean ± SE (standard error); the sample size was three (n = 3), and the level of statistical significance was set at ≤ 0.05. Superscript letters (a, b, and c) denote statistically significant values, and differences between these letters indicate statistically significant variations among the tested sample.

### Alterations in the rheological properties of flour depending on the content of germinated grain

**Fig 2:**
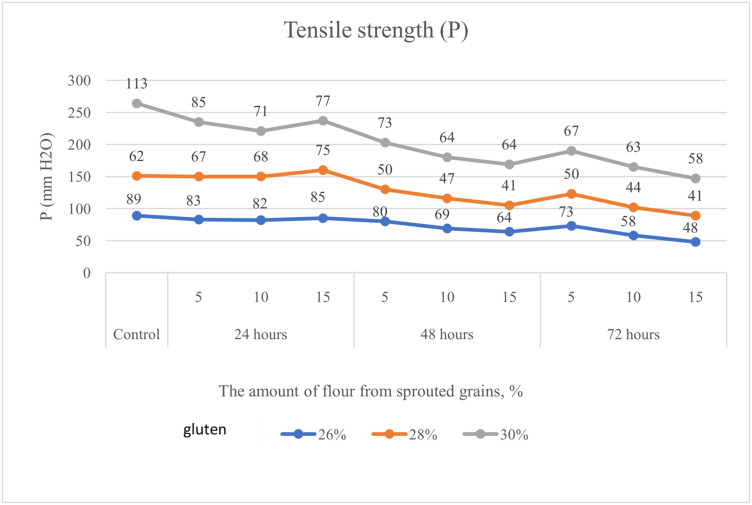
Alteration in dough tenacity (P) of flour with gluten contents of 26.0%, 28.0%, and 30.0%, supplemented with 5%, 10%, and 15% soft wheat grain germinated for 24, 48, and 72 hours.

The study of alterations in the rheological properties of soft wheat flour was conducted using samples with varying gluten contents (26.0%, 28.0%, and 30.0%) supplemented with 5%, 10%, and 15% of germinated wheat grain (germinated for 24, 48, and 72 hours). Results obtained using the alveograph consistently demonstrated a decline in key rheological parameters, including P (dough tenacity, mm H₂O), Ie (elasticity, %), and the P/L ratio (where L represents dough extensibility), as germination time increased. Furthermore, flour samples with different gluten contents exhibited a comparable deterioration in rheological properties. For instance, by the end of the experiment, dough tenacity (P) had decreased by 46%, 34%, and 48% in flours with 26.0%, 28.0%, and 30.0% gluten content, respectively (Fig 2).

**Fig 3:**
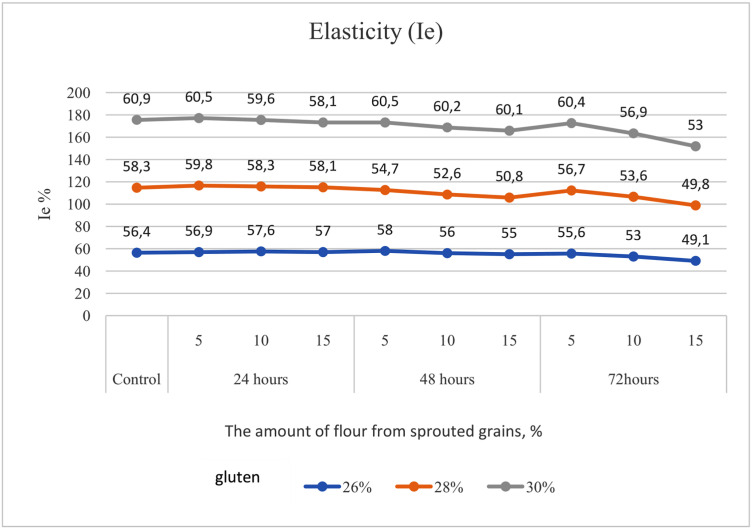
Alteration in elasticity (Ie) of flour with gluten contents of 26.0%, 28.0%, and 30.0%, supplemented with 5%, 10%, and 15% soft wheat grain germinated for 24, 48, and 72 hours.

The reduction in elasticity (Ie) in the same samples amounted to 13%, 15%, and 13%, respectively (Fig 3).

**Fig 4:**
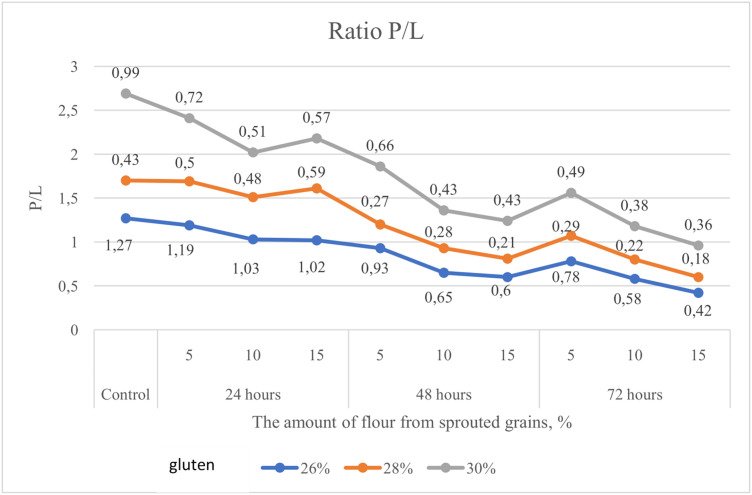
Alteration in the P/L ratio of flour with gluten contents of 26.0%, 28.0%, and 30.0%, supplemented with 5%, 10%, and 15% soft wheat grain germinated for 24, 48, and 72 hours.

The P/L ratio in the analyzed samples decreased as follows: by 66% in flour with a gluten content of 26.0%, by 58% in flour with 28.0%, and by 64% in flour with 30.0% gluten content (Fig 4).

**Fig 5:**
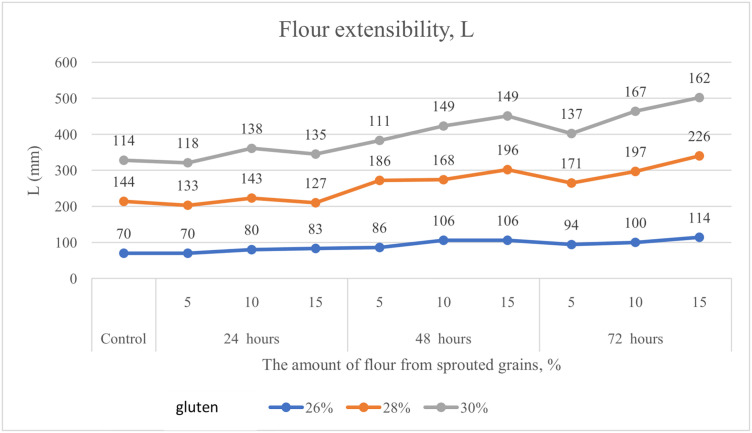
Alteration in dough extensibility (L) of flour with gluten contents of 26.0%, 28.0%, and 30.0%, supplemented with 5%, 10%, and 15% soft wheat grain germinated for 24, 48, and 72 hours.

At the same time, dough extensibility (L) (Fig 5) increased by 62%, 57%, and 42% in samples with gluten contents of 26.0%, 28.0%, and 30.0%, respectively.

**Fig 6:**
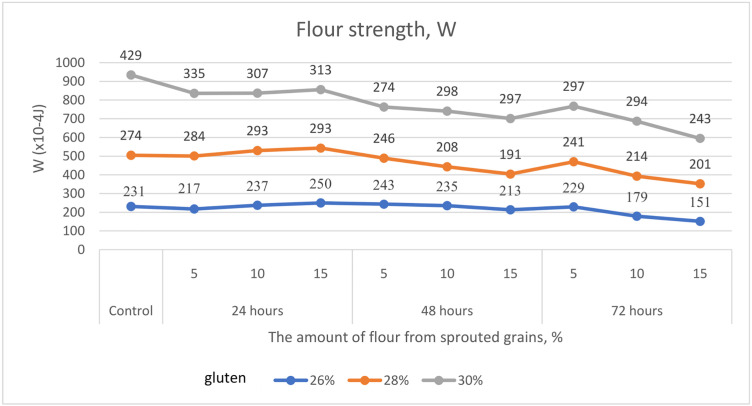
Alteration in flour strength (W) of samples with gluten contents of 26.0%, 28.0%, and 30.0%, supplemented with 5%, 10%, and 15% soft wheat grain germinated for 24, 48, and 72 hours.

All these alterations occurring in the germinated wheat grain were reflected in the flour strength (W) (Fig 6), which also decreased in the samples with gluten contents of 26.0%, 28.0%, and 30.0% by 35%, 27%, and 43%, respectively.

### Three-factor experiment

To determine the optimal germination conditions and proportion of germinated grain in the flour blend, three independent factors were selected: germination time, percentage of germinated grain, and gluten content. Evaluation criteria included flour strength, water absorption capacity, and falling number, which serve as sensitive indicators reflecting the combined effects of enzymatic and structural transformations occurring in germinated grain and their influence on the rheological and structural properties of the dough. As a result of the conducted experiments, values were obtained for W, WAC, and the FN in flour blends at varying levels of gluten content, germination time, and proportion of germinated soft wheat grains of the *Tәuelsizdik* variety (Table 3).

**Table 3:** Results of the three-factor experiment using soft wheat of the Tәuelsizdik variety.

Experiment No	Coded levels of factors			Actual levels of factors			W (×10 ⁻ ^4^ J)	WAC (%)	FN, sec
	*х*_*1*_	*х*_*2*_	*х*_*3*_	Gluten (%)	Time, h	P, %	*у*_*1*_	*у*_*2*_	*у*_*3*_
1	0	0	0	28	48	10	208 ± 0,17	55,0 ± 0,12	140 ± 0,23
2	−1	−1	0	26	24	10	237 ± 0,02	53,7 ± 0,17	175 ± 0,11
3	+1	−1	0	30	24	10	307 ± 0,12	55,1 ± 0,13	169 ± 0,19
4	−1	+1	0	26	72	10	179 ± 0,11	53,1 ± 0,05	89 ± 0,12
5	+1	+1	0	30	72	10	294 ± 0,09	54,2 ± 0,02	79 ± 0,17
6	−1	0	−1	26	48	5	243 ± 0,05	52,9 ± 0,0	178 ± 0,16
7	+1	0	−1	30	48	5	274 ± 0,19	54,2 ± 0,16	187 ± 0,23
8	0	0	0	28	48	10	209 ± 0,0	55,1 ± 0,11	138 ± 0,19
9	−1	0	+1	26	48	15	213 ± 0,11	53,2 ± 0,05	126 ± 0,02
10	+1	0	+1	30	48	15	298 ± 0,05	54,5 ± 0,02	129 ± 0,13
11	0	−1	−1	28	24	5	284 ± 0,07	54,3 ± 0,09	206 ± 0,17
12	0	+1	−1	28	72	5	241 ± 0,02	53,9 ± 0,05	109 ± 0,06
13	0	−1	+1	28	24	15	293 ± 0,21	55,6 ± 0,07	151 ± 0,09
14	0	+1	+1	28	72	15	243 ± 0,13	54,2 ± 0.01	72 ± 0,14
15	0	0	0	28	48	10	207 ± 0,11	54,9 ± 0,0	141 ± 0,18

X_1_ – Gluten content (coded values).

X_2_ – Germination time (coded values).

X_3_ – Percentage of germinated grain (coded values).

y_1_ – The power of flour (W).

y_2_ – Water Absorption Capacity (WAC).

y_3_ – Falling Number index (seconds).

Data are shown as mean ± standard error (SE); sample size n = 3; significance level was set at p ≤ 0.05.

### Effect of germinated grain on flour strength

In the study, flour strength varied from 179 × 10 ⁻ ⁴ J to 307 × 10 ⁻ ⁴ J. Based on the regression equation (1), a three-dimensional model was constructed, representing a response surface that illustrates the relationship between flour strength and the parameters of germinated grain.


$$y_{1}=3675.25-247.187\times 2-5.39583\times 2-24.0\times 3+4.75\times 21+0.047309\times 22+1.2\times 32$$ (1)

**Fig 7:**
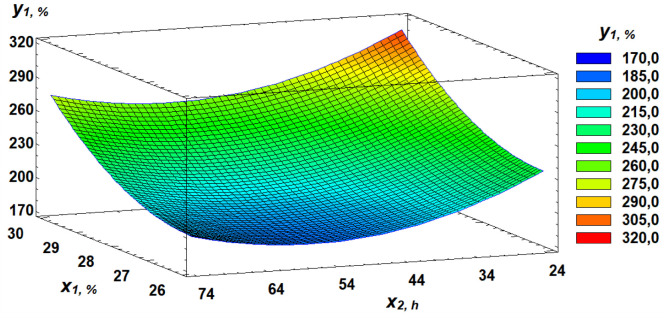
Response surface of the dependence of flour strength on gluten content and germination time.

**Fig 8:**
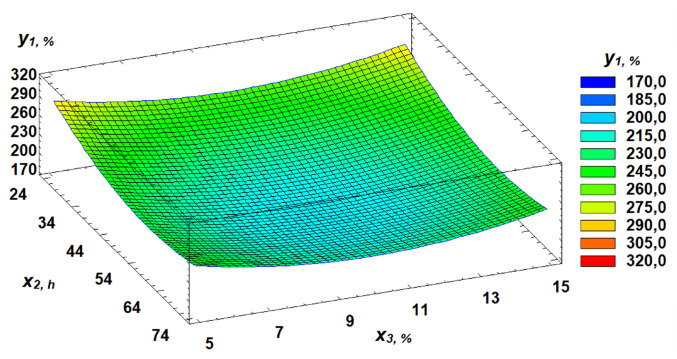
Response surface of the dependence of flour strength on germination time and proportion of germinated grains.

**Fig 9:**
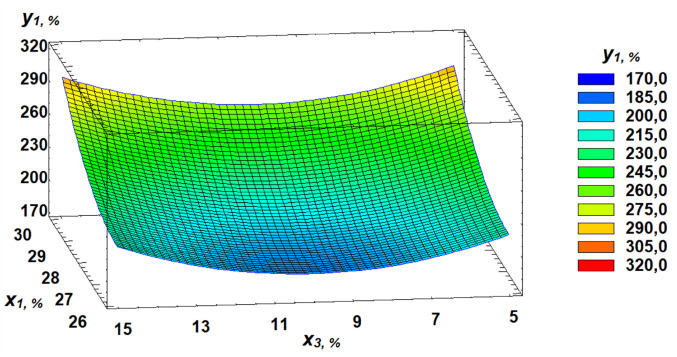
Response surface of the dependence of flour strength on gluten content and proportion of germinated grains.

The response surface plots visually demonstrate the dependence of flour strength on the selected factors (Figs 7–9).

### Effect of germinated grain on water absorption capacity

The analysis of variance (Table 4) for the WAC of flour demonstrated that six effects had p-values less than 0.05, indicating that they are statistically significant at the 95.0% confidence level.

**Table 4:** Analysis of Variance for Water Absorption Capacity.

Source	Sum of Squares	Degrees of Freedom (df)	Mean Square	F-Ratio	p-Value
x₁	3.25125	1	3.25125	124.89	0.0
x₂	1.36125	1	1.36125	52.29	0.0001
x₃	0.605	1	0.605	23.24	0.0013
x₁²	2.88137	1	2.88137	110.68	0.0
x₂x₃	0.25	1	0.25	9.6	0.0147
x₃²	0.611552	1	0.611552	23.49	0.0013
Residual	0.208269	8	0.0260337		
Total	8.996	14			

A regression equation was obtained describing the dependence of WAC on the germination factors of wheat grain.


$$y_{2}=-128,958+12,6495x_{1}+0,00364583x_{2}+0,479615x_{3}-0,220192x_{1}^{2}-0,00208333x_{2}^{2}-0,0162308x_{3}^{2}$$ (2)

**Fig 10:**
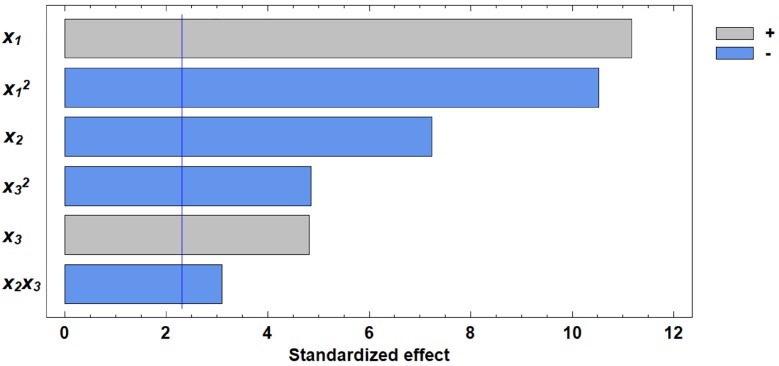
Pareto chart of standardized effects of independent factors on water absorption capacity.

**Fig 11:**
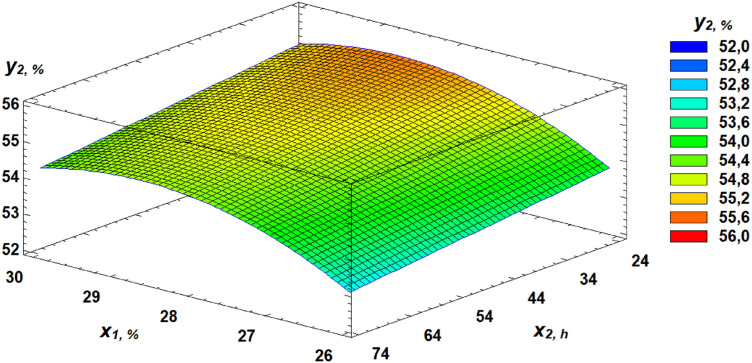
Response surface of the dependence of water absorption capacity on gluten content and germination time.

**Fig 12:**
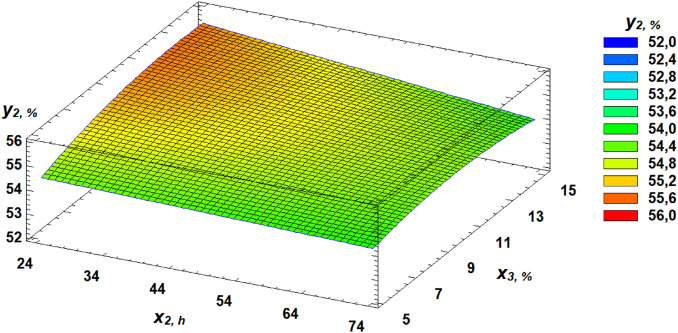
Response surface of the dependence of water absorption capacity on germination time and proportion of germinated grains.

**Fig 13:**
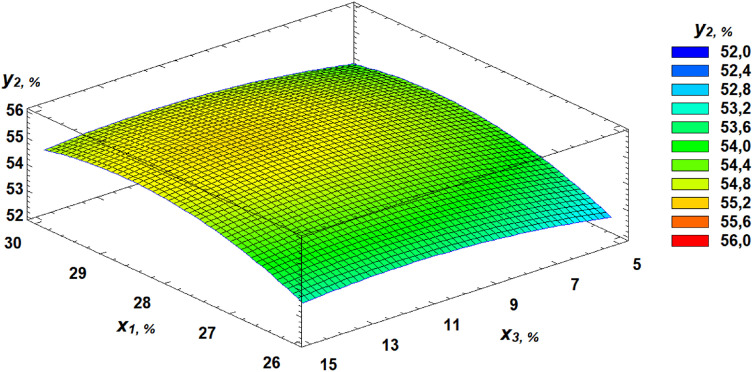
Response surface of the dependence of water absorption capacity on gluten content and proportion of germinated grains.

Analysis of the Pareto chart indicates that gluten content in the grain exerts the most substantial positive effect on water absorption capacity (WAC). Based on the derived regression equation, three-dimensional response surface models were constructed. These models represent planes that characterize the dependence of WAC on the germination parameters of wheat grain (Figs 10–13).

### Effect of germinated grain on the FN index

Similarly, the dependence of the FN on the parameters of germinated grain was established and is expressed by the following regression equation:


$$y_{3}\;=\;257,5\;+\;0,903846x_{2}\;-\;13,5115x_{3}\;-\;0,0285123x_{2}^{2}\;+\;0,423077x_{3}^{2}$$ (3)

**Fig 14:**
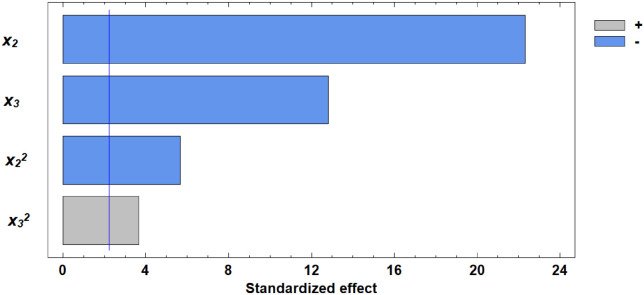
Pareto chart of standardized effects of independent factors on the falling number of flour.

Based on the derived regression equation (3), three-dimensional response surface models were constructed. These models represent planes that characterize the relationship between the falling number and the germination parameters of wheat grain. A Pareto chart of standardized effects provides a visual representation of the contribution of each independent factor (Fig 14).

Analysis of the Pareto chart indicates that germination time has the most significant negative impact on the falling number of flour.

**Fig 15:**
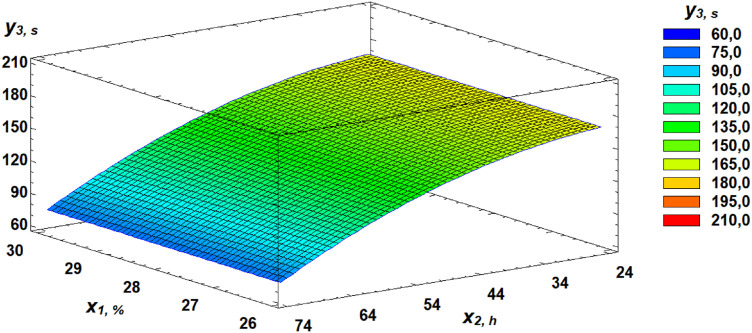
Response surface of the dependence of falling number on gluten content and germination time.

**Fig 16:**
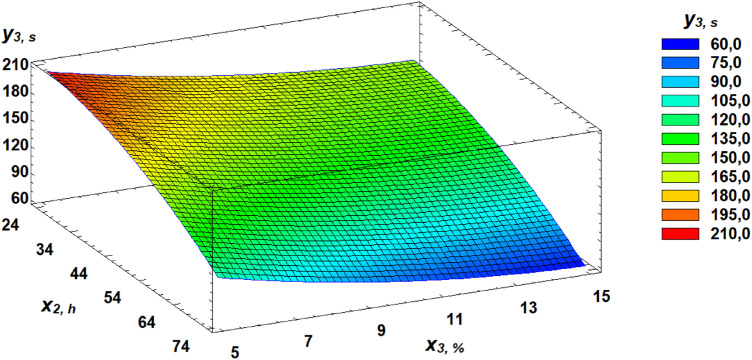
Response surface of the dependence of falling number on germination time and proportion of germinated grains.

**Fig 17:**
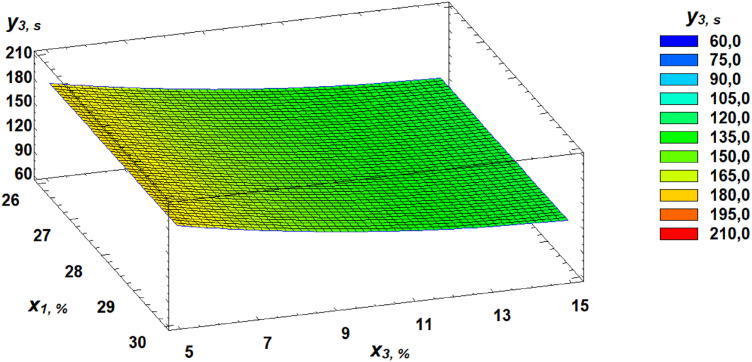
Response surface of the dependence of falling number on gluten content and proportion of germinated grains.

Further clarification of the effects of independent factors can be seen through the analysis of response surfaces, which are presented as three-dimensional plots illustrating the dependencies (Figs 15–17).

**Fig 18:**
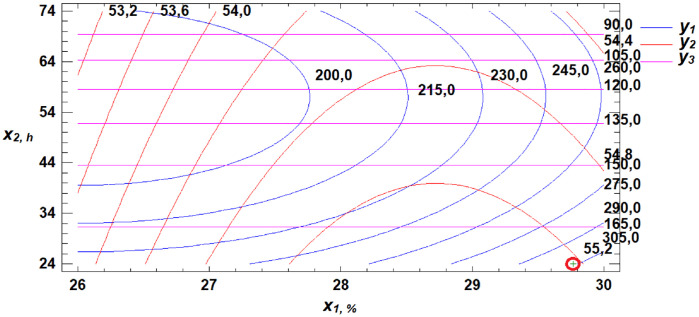
Section projections of the response surface illustrating the dependence of flour strength, water absorption capacity, and falling number on gluten content and germination time.

**Fig 19:**
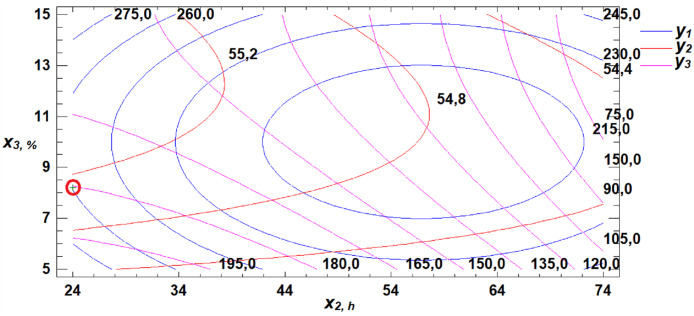
Section projections of the response surface illustrating the dependence of flour strength, water absorption capacity, and falling number on germination time and proportion of germinated grains.

**Fig 20:**
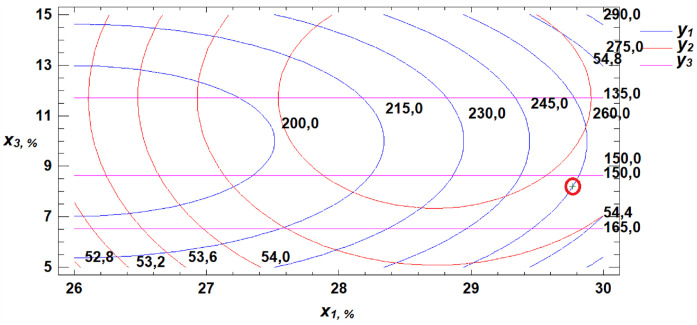
Section projections of the response surface illustrating the dependence of flour strength, water absorption capacity, and falling number on gluten content and proportion of germinated grains.

To determine the optimal parameters for the three obtained response functions, a multiple response optimization was performed. This procedure allows the identification of a combination of experimental factors that simultaneously optimize several response variables—in this case, W, WAC, and FN. The optimization is achieved by maximizing the desirability function, as illustrated in Figs 18–20.

Using the desirability function maximization method, combinations of factor levels were identified at which the optimum values of the selected technological properties of soft wheat grain—namely, flour strength, water absorption capacity, and falling number—are achieved (Table 5).

**Table 5:** Optimization of the Soft Wheat Grain Germination Process Using the Desirability Function.

Factors	Minimum	Maximum	Optimum	Response	Optimum Value
Gluten content, %	26.0	30.0	29.8	Flour strength (W × 10 ⁻ ⁴ J)	307.6
Germination time, h	24.0	72.0	24.0	Water absorption capacity, %	54.9
Proportion of germinated grain, %	5.0	15.0	8.2	Falling number, s	180.4

## Discussion

The potential use of germinated soft wheat grain, which differs significantly from sound grain, for technological purposes presents considerable interest. This is particularly relevant for soft wheat varieties developed through Kazakhstani breeding programs and cultivated in the country’s major grain-producing regions, which frequently experience unfavorable harvesting conditions. The findings of the present study demonstrate that germinated grain can be utilized, provided it meets the quality indicators defined in the conducted experiments.

When working with germinated grain, certain considerations must be taken into account. According to Kaur, Gasparre [23], based on morphological evaluation, wheat grains germinated for 12–24 hours are classified as germinated, whereas grains germinated for 36 hours are considered severely germinated, as the broken sprout extends beyond the embryo outline. Our data clearly indicate that the degree of germination after 24 hours is sufficient for classifying the grain as germinated (Fig 2). Notably, germinated wheat grains that have lost their sprouts are visually indistinguishable from sound grains. Therefore, only physicochemical indicators can serve as constraints in determining whether germinated grain can yield quality milling products.

During germination, enzymatic activity in the grain increases, which, as reported by several studies [1,36,37], leads to the degradation of proteins, lipids, and carbohydrates. They suggest that the observed increase in protein content during germination may result from the release of free nitrogen due to enzymatic protein degradation. In contrast, Kaur, Gasparre [23] found that germination does not significantly affect protein content. In our study, a slight increase in protein content was observed, which is in agreement with Badawy, Al-Dalain [36] and Yang, Yin [37], as the result of increased free nitrogen. However, free nitrogen does not contribute to gluten formation. As such, despite the typical correlation between protein and raw gluten content in sound wheat grain, our findings showed a continuous decline in gluten content—initially by 2%, and more substantially by 17% at the final stage—an observation not previously reported.

Zhang, Wang [38] reported a decrease in dry gluten content, attributing it to the degradation of high molecular weight proteins and the subsequent increase in soluble proteins, which do not support gluten formation. Therefore, the protein-gluten relationship observed in sound grain is disrupted in germinated grain and requires further investigation.

There is also differing opinion regarding starch, considered the most critical component during germination. While Kaur, Gasparre [23] observed a decline in starch content only after 36 hours of germination, our results, consistent with Verma, Kumar [39], showed starch degradation only after 72 hours. Mendoza Moreno, Palma-Rodríguez [1] attribute this to starch hydrolysis into various sugars during germination. We believe that the rate of starch degradation depends directly on the initial quality of the grain, and comprehensive studies involving diverse soft wheat samples are needed to establish consistent patterns.

The FN, which reflects α-amylase activity, is considered one of the most informative quality indicators for germinated wheat grain. Okuyama, Riede [40] reported a negative linear regression between the percentage of germination and FN in wheat genotypes exposed to pre-harvest sprouting. Hull, Swanepoel [41] and Okuyama, Riede [40] found that reduced FN is associated with increased α-amylase activity, which hydrolyzes starch molecules, significantly lowering dough viscosity, reducing loaf volume, and producing sticky crumb in bread.

Cardone, Grassi [42] observed up to a 600-fold increase in α-amylase activity in whole wheat flour germinated for 48 hours at 20°C. Similar findings were reported by Zhang, Pritchard [43–46]. These results confirm that longer germination leads to increased α-amylase activity and structural breakdown of starch, reducing its ability to form a viscous gel and thereby lowering FN.

In our study, FN declined by 73% from an initial value of 233 seconds within just 24 hours of germination, drastically diminishing its technological suitability. Pareto chart analysis of the factorial experiment results showed that germination time had the most negative effect on FN (Fig 16). The established regression equation (3) describing the relationship of FN with gluten content, germination time, and the proportion of germinated grain allows for FN prediction without experimental testing.

Equally important are the rheological indicators of soft wheat grain. According to Hadnađev, Torbica [47], these parameters are essential for evaluating flour quality, as they help monitor molecular structure, mechanical properties, composition, and predict the final product’s quality. Our study considered the following rheological parameters in flour with varying percentages of germinated grain: P – dough tenacity, Ie – elasticity, L – extensibility, and the P/L ratio. Banu, Patraşcu [13] reported that control samples had significantly higher rheological values compared to flour blends containing germinated grain (Figs 3–7). Mendoza Moreno, Palma-Rodríguez [1] found that 12-hour germination reduced dough resistance (from 0.28 N to 0.11 N) and extensibility (from 10.19 mm to 4.15 mm). Cauduro, D’Almeida [45] also observed that germination time and replacement level of sound grain with germinated grain decreased water absorption, dough stability, development time, and resistance to extension.

These effects can be attributed to enzyme activation and structural changes in grain proteins and starch. Proteolytic enzymes degrade gluten networks, shortening polypeptide chains in glutenin and gliadin, reducing molecular weight, and weakening gluten structure [13]. Starch degradation and accumulation of soluble sugars reduce dough viscosity and further enhance plasticity.

In our study, dough tenacity decreased by an average of 43%, elasticity dropped by 14%, while extensibility almost doubled, increasing by 54% (Fig 6). All these changes in germinated wheat grain affected flour strength, which declined on average by 35% (Fig 7). The most significant positive influence on flour strength was gluten content (Fig 8), consistent with Guo, Wang [48], who stated that dough rheology is primarily determined by gluten content. However, in line with the findings of Navarro, Losano Richard [49], germination time had the most pronounced negative effect (Fig 9). We additionally examined the effect of gluten content on flour strength. The derived regression equation (1) allows for prediction of W based on known factor levels.

Another important rheological property evaluated in our study was the WAC of flour. Kaur and Gill [50] suggest that the decrease in WAC observed during germination could be due to protein depolymerization from protease activity. Luciński and Adamiec [51] also noted that proteolytic activity hydrolyzes gluten and partially breaks down high molecular weight proteins into simple ones, adversely affecting dough rheology and reducing WAC. Our findings showed that gluten content had the most positive effect on WAC, while increased germinated grain content and germination time had negative effects. These relationships are expressed in regression equation (2).

One of the main limitations of the present study is the use of a single soft wheat variety. While this approach allowed for controlled experimental conditions and minimized the influence of genetic variability, it may limit the generalizability of the findings across different wheat cultivars. The decision to focus on a single variety was intentional, aimed at isolating the effects of germination duration and the proportion of germinated grain in flour blends without confounding varietal differences. However, the outcomes may differ with other soft or hard wheat genotypes, given their distinct technological and biochemical characteristics. Future studies should therefore aim to validate these findings using multiple wheat varieties to enhance the robustness and applicability of the results across broader agronomic and processing contexts.

## Conclusion

A review of the literature reveals that previous studies offered limited insight into how different germination conditions affect the technological properties of germinated wheat flour. Experimental results allowed us to identify optimal ranges for key technological indicators. Maximum flour strength was observed at 30% gluten content, 24 hours of germination, and 15% germinated grain. Optimal WAC was achieved at 28.7% gluten content, 24 hours of germination, and 13.2% germinated grain. The optimal falling number zone was observed at 28% gluten, 24 hours of germination, and 5% germinated grain. Based on the developed mathematical model, we determined the optimal germination parameters for soft wheat grain to be: 29.8% gluten content, 24 hours of germination, and 8.2% germinated grain. At these values, the resulting flour meets the optimal technological performance criteria.

## Supporting information

S1 Table Raw data. Values.
(ZIP)
