# Supplementary material for: The influence of soft wheat grain germination on its technological properties
Source: PLoS One. 2025 Aug 13;20(8):e0329975. doi: 10.1371/journal.pone.0329975 (PMC12349700; doi:10.1371/journal.pone.0329975)
Supplement: S1 Table — Values. (ZIP) [file pone.0329975.s001.zip › Dataset/Table.docx]

| Kernel Weight | 1 | 2 | 3 | Mean value |
| --- | --- | --- | --- | --- |
| 24 h | 25,9 | 26,2 | 26,12 | 26,07±0.16 |
| 48 h | 25,62 | 25,35 | 25,54 | 25,50±0.14 |
| 72 h | 24,2 | 24,16 | 24,48 | 24,28±0.18 |

Data of wheat "Tauelsizdik"

| Experiment No | Mean value FN | Repetitions | | |
| --- | --- | --- | --- | --- |
| 1 | 91±0 | 91 | 91 | 91 |
| 2 | 120,3±0.58 | 121 | 120 | 120 |
| 3 | 121,3±1.53 | 123 | 121 | 120 |
| 4 | 113±2 | 115 | 113 | 111 |
| 5 | 98±8 | 90 | 98 | 106 |
| 6 | 129±2 | 131 | 129 | 127 |
| 7 | 133,3±2.52 | 136 | 133 | 131 |
| 8 | 135±1 | 134 | 135 | 136 |
| 9 | 122±1 | 123 | 122 | 121 |
| 10 | 129±2 | 131 | 129 | 127 |
| 11 | 103,3±1.53 | 102 | 103 | 105 |
| 12 | 127±0 | 127 | 127 | 127 |
| 13 | 123±1 | 122 | 123 | 124 |
| 14 | 92±1 | 91 | 92 | 93 |
| 15 | 117,3±0.58 | 118 | 117 | 117 |
| 16 | 84,3±3.51 | 88 | 84 | 81 |

Data of wheat "Tauelsizdik"

| № | Moisture, % | Protein, % | Gluten, % | W, J | Sedimentation, mL | Test Weight, hL |
| --- | --- | --- | --- | --- | --- | --- |
| Control | 13,38 | 13,6 | 28,7 | 235 | 38 | 766 |
| Control | 13,21 | 13,6 | 28,4 | 234 | 38 | 775 |
| Control | 13,25 | 13,5 | 28,7 | 236 | 38 | 772 |
| Mean value | 13,28±0,09 | 13,50±1,0 | 28,6±0,3 | 235±1 | 38±0 | 771±9 |
| 24 h | | | | | | |
| 1. | 11,93±0,20 | 13,73±0,12 | 26,16±0,21 | 174±1,0 | 35±0,0 | 637±4,16 |
| 1.1 | 11,75 | 13,8 | 26,4 | 175 | 35 | 634 |
| 1.2 | 12,14 | 13,8 | 26,1 | 174 | 35 | 636 |
| 1.3 | 11,91 | 13,6 | 26,0 | 173 | 35 | 642 |
| 48 h | | | | | | |
| 2. | 11,62±0,05 | 13,9±0,1 | 25,9±0,1 | 163±3,5 | 35±0,0 | 632±3,5 |
| 2.1 | 11,63 | 14,0 | 26,0 | 165 | 35 | 635 |
| 2.2 | 11,67 | 13,9 | 25,9 | 159 | 35 | 628 |
| 2.3 | 11,58 | 13,9 | 25,9 | 166 | 35 | 634 |
| 72 h | | | | | | |
| 3. | 10,0±0,035 | 13,9±0,25 | 23,6±0,3 | 118±10 | 31±0,5 | 596±2,5 |
| 3.1 | 10,04 | 14,1 | 23,3 | 108 | 31 | 594 |
| 3.2 | 10,0 | 14,1 | 23,9 | 128 | 32 | 599 |
| 3.3 | 9,97 | 13,6 | 23,8 | 118 | 32 | 596 |
